# Dietary compounds slow starch enzymatic digestion: A review

**DOI:** 10.3389/fnut.2022.1004966

**Published:** 2022-09-15

**Authors:** Chengdeng Chi, Miaomiao Shi, Yingting Zhao, Bilian Chen, Yongjin He, Meiying Wang

**Affiliations:** ^1^College of Life Sciences, Fujian Normal University, Fuzhou, China; ^2^College of Food and Biological Engineering, Zhengzhou University of Light Industry, Zhengzhou, China; ^3^Center for Nutrition and Food Sciences, The University of Queensland, Queensland Alliance for Agriculture and Food Innovation, Brisbane, QLD, Australia; ^4^College of Food Science, Fujian Agriculture and Forestry University, Fuzhou, China; ^5^School of Engineering, University of Guelph, Guelph, ON, Canada

**Keywords:** starch digestion, dietary compounds, starch structure, enzyme activity, nutrition

## Abstract

Dietary compounds significantly affected starch enzymatic digestion. However, effects of dietary compounds on starch digestion and their underlying mechanisms have been not systematically discussed yet. This review summarized the effects of dietary compounds including cell walls, proteins, lipids, non-starchy polysaccharides, and polyphenols on starch enzymatic digestion. Cell walls, proteins, and non-starchy polysaccharides restricted starch disruption during hydrothermal treatment and the retained ordered structures limited enzymatic binding. Moreover, they encapsulated starch granules and formed physical barriers for enzyme accessibility. Proteins, non-starchy polysaccharides along with lipids and polyphenols interacted with starch and formed ordered assemblies. Furthermore, non-starchy polysaccharides and polyphenols showed robust abilities to reduce activities of α-amylase and α-glucosidase. Accordingly, it can be concluded that dietary compounds lowered starch digestion mainly by three modes: (i) prevented ordered structures from disruption and formed ordered assemblies chaperoned with these dietary compounds; (ii) formed physical barriers and prevented enzymes from accessing/binding to starch; (iii) reduced enzymes activities. Dietary compounds showed great potentials in lowering starch enzymatic digestion, thereby modulating postprandial glucose response to food and preventing or treating type II diabetes disease.

## Introduction

Starch which is a polysaccharide composed of linear chains (amylose) or branched chains (amylopectin) is a major source of energy in the human diet. Starch digestion is accomplished by two type of enzymes in human gastrointestinal tract (GIT): (i) salivary and pancreatic α-amylases and (ii) intestinal brush border glucoamylases, maltase-glucoamylase, and sucrase-isomaltase (1). Amylase digests amylose into maltose subunits (disaccharide) and amylopectin into branched chains (i.e., dextrins). Both maltose and dextrins are digested by enzymes located in intestinal brush border, which in turn produced glucose. The glucose released from starch is subsequently absorbed in the intestine and hydrolyzed to produce adenosine triphosphate or stored in animals as the polysaccharide glycogen. Accordingly, starch-based diets are commonly the main foods that provide the necessary energy. However, rapid digestion of starch contributes to postprandial hyperglycaemia, which in turn possibly results in an impaired insulin secretion and the incidence of chronic diseases such as obesity and type II diabetes (2, 3). Slowing starch enzymatic digestion in the GIT is of great interest in preventing the incidence of chronic diseases.

Enzymatic reactions consist of three steps: diffusion of enzymes to the solid surfaces, absorption/binding, and catalysis (1). Regarding to starch enzymatic digestion, there are two factors influencing the extent and rate of starch digestion: (i) barriers that slow down or prevent digestive enzymes from accessing/binding to starch and (ii) starch structural features that limit enzyme action after initial binding (4). Starch structuration on fine structure, helical structure, crystalline structure, lamellar structure, short-range ordered structure, and nanoscale aggregate structure significantly slowed enzymes binding with starch and reduced enzymes catalyzation toward starch (5–13). It has been summarized that slowly digestible starch (SDS) was the fraction with high α-1,6 linkages, short branch chains [degree of polymerization (DP) <13], long chains with DP 25–36, or imperfect helical and crystalline structures, while the resistant starch (RS) was the fraction rich in high amylose content, double helix-promoting chains with DP *ca*. 12–24 and DP ≥ 37, along with some chains with DP 25–36, perfectly-packed double helices and crystalline structures, V-type crystals, or densely-packed crystalline lamellae and more ordered reassembled aggregate structures (8). According to previous studies (14–19), starch digestion was affected not only by its intrinsic structures but also by the interactions between starch and dietary non-starchy compounds and between digestive enzymes and non-starchy foods. At present, many reviews have indicated dietary compounds such as polyphenols, lipids, and non-starchy polysaccharides significantly affected starch digestion (15–20). However, the effects of dietary compounds such as cell walls, protein, lipids, non-starchy polysaccharides, and polyphenols on starch digestion and their underlying mechanisms have been not systematically summarized yet.

Therefore, this review provided a survey of the latest developments on dietary strategies for slowing starch enzymatic digestion, with a particular focus on the mechanisms underlying the modulation of starch digestion. Future perspectives regarding the dietary strategies for the control of starch digestion will be proposed. This review can provide better insights into the modulation of starch enzymatic digestion through complexation with dietary compounds.

## Cell walls slow starch digestion

**Figure 1:**
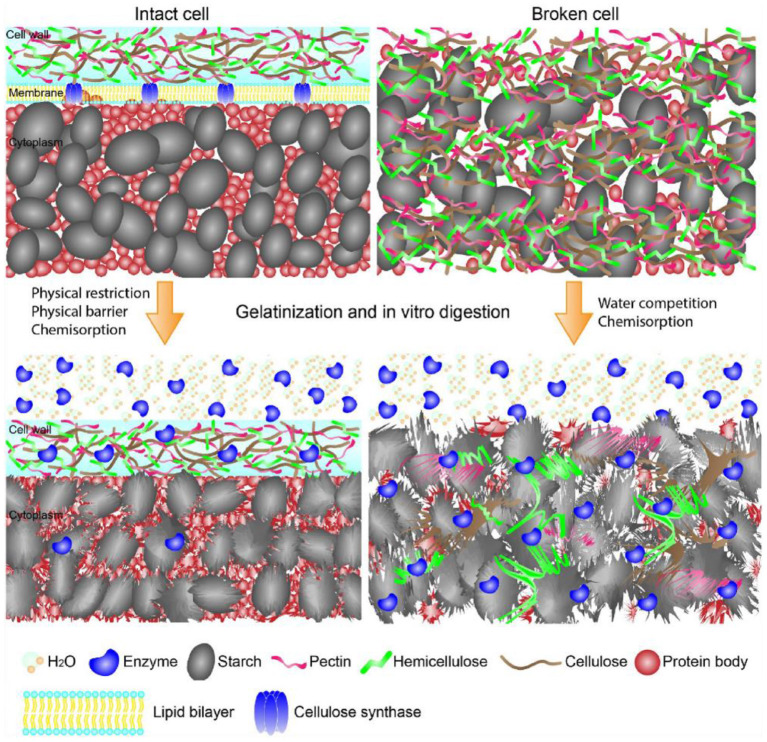
Schematic showing the structures of starch-containing intact and broken cells during gelatinization and *in vitro* digestion. The intact cell walls limited enzymes and water diffusion onto starch, while the intact cell walls yielded starch complete gelatinization and enzymes diffusion onto starch surface. The graph is collected from Li et al. (21).

The basic architecture of plant cell wall is shown in Figure 1. Plant cell walls are cellulose-based assemblies containing cellulose and non-cellulosic polysaccharides (e.g., pectin, xyloglucans, heteroxylans, and β-glucans), lignin and some proteins (20, 21). Cellulose fibrils assembled and served as scaffold filling with amorphous non-starchy polysaccharides (20). While the filling non-starchy polysaccharides prevented aggregation and collapse of the cellulose/hemicellulose network, the interactions of the non-starchy polysaccharides significantly contributed to the density and porosity of cell walls and in turn determined the permeability of hydrolases through the cell walls (22, 23).

Effects of cell walls on starch gelatinization and enzyme-starch interaction are shown in Figure 1 (21). Cell walls entrapped starch granules, thereby limiting the enzymatic digestion through the physical barriers (24). In addition, non-starchy polysaccharides-rich cell walls of starchy foods retained their intact structures during food processing, and in turn significantly slowed starch enzymatic digestion (25, 26). Non-starchy polysaccharides were not digested in the GIT due to the lack of corresponding enzymes. Therefore, the cell walls of processed foods provided physical barriers for enzymes diffusion to starch and hydrolyzation of starch molecules. Li et al. suggested that the integrity of cell walls of pulse significantly affected starch digestion (27). Food processing yielded disorganization of cell walls, which in turn increased cell wall permeability and facilitated enzyme diffusion through the cell walls along with increased starch enzymatic digestion (27–31). Treatments with a higher temperature or a longer time promoted starch swelling, weakening the physical barriers, and increasing the degree of process-induced cell wall permeability, which in turn increased starch enzymatic digestion (29, 31). Although decreasing cell intactness has been shown to increase the rate but not the extent of starch digestion (26), most of studies indicated that the increase in cell permeability slowed the rate and reduced the extent of starch digestion *in vitro* (24, 25, 28–30, 32–34).

Li et al. indicated that enzymatic digestion of starch granules entrapped within cell walls in pulses depended on both the intactness of cell walls as well as the ordered structures of pulses after food processing (27). Cell walls not only limited enzymes diffusion onto starch molecules, but also delayed starch gelatinization, retarded starch granules swelling and leaching of starch molecules. Accordingly, ordered structures (e.g., helical structures, crystalline structures, granular form) of starch granules within cell walls retained as shown in Figure 1 (21, 27–29, 32, 34, 35). The retained starch ordered structures significantly slowed starch enzymatic digestion (7, 8). Thus, the cell walls slowed starch digestion through retarding the disruption of starch ordered structures during food hydrothermal processing.

In addition to the roles of cell walls on starch ordered structures, cell walls also bound with enzymes and affected hydrolyzation activities of the enzymes (25, 34). The non-catalytic binding of amylase on cell walls limited enzymes diffusion onto starch molecules, which reduced the amylolysis of starch within intact cells (25, 34). Starchy foods like pulses contained α-amylase inhibitors such as tannins, lectins and other proteinaceous inhibitors (36). Li et al. reported that both soluble and insoluble components from pulse cells showed a significant inhibition (*ca*. 3–15%) on enzymes activities, thereby slowing the enzymatic digestion of starch within pulse cells (34).

## Proteins slow starch digestion

Protein is one of the most important compounds in foods systems. It has been summarized that proteins could non-covalently interact with starch through hydrogen bonding, hydrophobic interactions, electrostatic forces, ionic interactions, and van der Waals force (17). Endogenous rice protein interacted with rice starch significantly lowered starch digestion extent through reducing swelling of starch granules and suppressing the accessibility of enzymes to starch granules (37). Potato protein isolate interacted with starch and in turn restricted starch disorganization and reduced starch digestion extent (38). Denatured plant proteins interacted with starch through hydrogen bonds and electrostatic interactions and restricted starch hydration and enzymatic cleavage (39). Whey protein interacted with starch to form starch-protein assemblies, which significantly increased starch short-range ordered structure while lowering starch digestion extent (40). Enzymatically hydrolyzed (the combination of pepsin and pancreatin) rice protein interacted with rice starch to promote the formation of V-type crystals and lowered starch digestion extent (41). In addition to the role of proteins on structures of pure starch and starch-protein assemblies, some proteins bound with enzymes and lowered enzymes activities and finally reduced the digestion rate and extent of starch (41–43). Water soluble barley proteins bound with α-amylase, which reduced α-amylase activity and slowed starch digestion rate and lowered starch digestion extent (42). Rice proteins hydrolyzed by pepsin and pancreatin bound with α-amylase, and in turn, inhibited α-amylase activity [the IC_50_ value (the half maximal inhibitory concentration) was in the range of 1.75–2.15 mg/mL] and lowered starch digestion extent (41). The activity of α-amylase was decreased greatly from 0.42 to 0.07 units by native gluten pepsin-hydrolyzed gluten (43).

Although the effects of protein structures on starch digestion in foods systems has not been resolved yet, it can be preliminarily concluded that dietary protein has a strong ability to mitigate starch enzymatic digestion. Dietary proteins affected starch enzymatic digestion *via* different pathways: (i) proteins acted as physical barriers and restricted the interaction of enzymes with starch molecules (37); (ii) proteins interacted with starch and restricted starch swelling and disorganization during hydrothermal treatment, which increased ordered structures and blocked the binding sites of starch molecules for digestive enzymes (39, 41); (iii) proteins or their hydrolysates interacted with starch to form ordered structures which were slowly digestible or not digestible (40, 41); (iv) proteins bound with α-amylase and lowered α-amylase activities and starch digestion extent (41, 42).

## Lipids slow starch digestion

Lipids are the important hydrophobic dietary compounds in foods. In starch-containing foods systems, lipids tended to interact with starch through hydrophobic interaction and formed starch-lipid inclusion complexes or starch-lipid-protein complexes (18). According to Dhital et al. (4), the access of enzymes to the glucosidic bonds in the substrate is a key factor affecting starch enzymatic digestion. The interaction between lipids and starch significantly changed the torsion angles of the glucosidic bonds, forming the starch helical structure and in turn affecting the binding activity of the amylolytic enzymes (18). The intact structures of starch-lipids inclusion complexes did not favor the formation of enzyme-substrate complexes (44). Accordingly, starch-lipids inclusion complex was classified into type-5 RS (45). Promoting the formation of starch-lipids inclusion complexes significantly lowered starch digestion extent.

Starch structures, lipid type and structures, and the preparation conditions significantly affected the formation of starch-lipid inclusion complexes. Amylose is much easier to interact with lipids compared with amylopectin. Starches with higher amylose content formed more starch-lipids complexes compared with less amylose-containing starches (46, 47). Debranching using pullulanase or isoamylase increased amylose content, which favored the formation of starch-lipid complexes and reduced starch digestion extent in a higher magnitude (48). A suitable polymerization of amylose is required for the formation of starch-lipid complexes (44, 49, 50). Increasing the chain length of amylose favored the formation of starch-lipid inclusion complexes (49), while a very long amylose hampered the formation of starch-lipid inclusion complexes (50).

Increasing lipids concentration favored the interaction of lipids with starch, thereby lowering starch digestion in a higher magnitude (51). However, lipids might self-assemble at a high concentration and reduce the content of starch-lipid inclusion complexes formed during the reaction (52). Free fatty acids formed starch-lipid inclusion complexes as a function of concentration (51, 53). Monoglycerides and phosphatidylcholine could also interact with starch and formed starch-lipids inclusion complexes (54, 55). Since monoglycerides had higher solubility in water compared with fatty acids, they were more likely to interact with starch and formed more starch-lipid inclusion complexes (56). However, diglycerides (e.g., dipalmitate glycerol) and triglycerides (e.g., tripalmitate glycerol) could not form inclusion complexes with starch because of their steric hindrance and low solubility in water (56). By reducing the carbon chain length of free fatty acids, the complexation index of fatty acids increased and the content of starch-lipid inclusion complexes significantly increased (57). The degree of unsaturation also affected the formation of starch-lipid inclusion complexes and starch digestibility. Fatty acids with a lower unsaturation could formed more inclusion complexes with starch, thereby greatly decreasing starch digestion extent (58, 59).

Proteins in food systems affected the formation of starch-lipid inclusion complexes. β-lactoglobulin favored lipids (e.g., fatty acids and monoglyceride) dissolution in water and promoted lipid-starch entanglement (58, 60–62). Notably, β-lactoglobulin promoted fatty acids which had a shorter length and lower unsaturation interaction with starch and formed starch-fatty acids-β-lactoglobulin complexes, while β-lactoglobulin in the binary system of starch, β-lactoglobulin, and monoglyceride rather promoted the formation of starch-monoglyceride complexes (61). Fatty acids contained carboxyl groups and might behave negatively in food systems, allowing fatty acids to interact with starch through hydrophobic interactions and with proteins through electrostatic interactions (63–65). However, monoglyceride is neutrally charged and cannot electrostatically bridge the formation of the starch-monoglyceride-β-lactoglobulin (61). In addition to the lipid types, protein types also affected the formation of starch-lipids inclusion complexes (66). Whey protein isolate and A-type gelatin promoted linoleic acid interaction with starch because of their emulsifiability. A-type gelatin showed a weaker ability to promote the formation of starch-linoleic acid inclusion complexes, which was attributed to the fact that A-type gelatin which had an isoelectric point higher than 7.0 might compete with starch for linoleic acid and reduce the accessibility of linoleic acid to starch hydrophobic cavity (66).

During the preparation of starch-lipid inclusion complexes, the temperature, complexation time and modes, pH, NaCl, and cooling rate significantly affected the formation of the complexes (67–71). A higher temperature and a longer time of the complexation, better-defined structures of the inclusion complexes had (67, 68, 71, 72). Regarding the complexation of swelled normal corn starch granules with lauric acid, the modes of adding lauric acid to the starch slurry [adding the lauric acid to the heated starch suspension (method I) or adding the lauric acid to the starch suspension and then heating (method II)] affected the content of starch-lauric acid inclusion complexes formed during the reaction (73). The method I was more beneficial to the formation of starch-lauric acid inclusion complexes than that of method II, because the lauric acid interacted with starch granules on surface, thereby inhibiting the migration of lauric acid into interior starch granules to form the complexes (73). A system with a higher pH promoted the formation of starch-lauric acid inclusion complexes and starch-lauric acid-β-lactoglobulin complexes, which was attributed to the greater solubility of lauric acid and higher leaching of amylose in the system (69). The presence of NaCl promoted the formation of starch-fatty acid inclusion complexes due to the improved solubility of fatty acids in NaCl-containing aqueous medium (70). The cooling rate of starch paste affected starch mobility during the cooling, which significantly affected structures of starch-lipid inclusion complexes (74, 75). At a higher cooling rate, amylose reorganized faster and more lipids could be entrapped into amylose hydrophobic cavity to form starch-lipid inclusion complexes (71).

## Non-starchy polysaccharides slow starch digestion

Non-starchy polysaccharides affected starch gelatinization, the viscosity of starch paste, starch reorganization, and enzymes activities, which affected starch digestion by different modes. Polysaccharides such as chitosan, guar gum, and xanthan interacted with starch granules and lowered starch swelling and amylose leaching during hydrothermal treatment (76–80). Pectin, κ-carrageenan, guar gum, arabic gum, pullulan, *Cordyceps* polysaccharides, *Mesona chinensis* polysaccharides, agar, xanthan gum and konjac glucomannan restricted starch disruption during the hydrothermal treatment and interacted with starch to form ordered structures, and in turn, lowered starch digestion extent (77, 78, 81, 82). Xanthan gum, guar gum, pectin, and konjac-glucomannan might interact starch and form physical barriers around starch molecules, reducing enzymes accessibility to starch molecules and lowering starch digestion extent (83, 84).

Due to the interaction between starch and non-starchy polysaccharides, the viscosity of starch suspension which complexed with xanthan gum, guar gum, konjac glucomannan, pectin, and chitosan significantly increased (85, 86). The increased viscosity of starch suspension in turn retarded enzymes diffusion onto starch surface, leading to a significant reduction in starch digestion extent (85, 86). Other soluble fibers including locust bean gum, fenugreek gum, fenugreek gum, and soy soluble polysaccharide also limited enzymes diffusion toward starch molecules and retarded glucose liberated from the starch-polysaccharide systems (86). It seems that starch digestion rate and extent could be controlled through modulating the viscosity of the starchy food systems.

The interaction between starch and non-starchy polysaccharides also significantly affected properties of matrix structures formed by starch and non-starchy polysaccharides (82, 87–89). Agar, xanthan gum and konjac glucomannan in starch pastes significantly promoted the formation of gel-like matrix structures, and in turn, lowered starch digestion rate and extent (87). The interaction between starch and non-starch polysaccharides and the increased gel rigidity of the matrix were the key factors affecting starch enzymatic digestion (87). *Mesona chinensis* polysaccharides also interacted with starch and significantly promoted the formation of a more ordered structure of blended systems of starch and *Mesona chinensis* polysaccharides, which remarkably lowered starch digestion rate and extent (82, 88, 89). Comparing with xanthan, guar, locust bean gum, and agar, starch-*Mesona chinensis* polysaccharides complexes had better-defined gel structures and the *Mesona chinensis* polysaccharides were found to be the most effective polysaccharides in reducing wheat starch digestion (88).

Starch digestion was controlled not only by starch ordered structures and food viscosity, but also by the activities of enzymes. Pectin bound with pancreatic amylase to reduce amylase activity, resulting in slower starch enzymatic digestion (90). Polysaccharides from oat (*Avena sativa L*.), *Camellia oleifera* Abel. fruit hull, oolong tea, shaddock (*Citrus aradise*), *Coriolus versicolor* LH1, mulberry fruit, pumpkin (*Cucurbita moschata*) fruit, fermented puerh tea, green tea flower, corn silk, *Acacia tortilis gum* exudate, Chinese traditional medicine Huidouba, *Mallotus furetianus*, hemp (*Cannabis sativa L*.), *Fagopyrum tartaricum*, blackberry fruit, *Rosa roxburghii* Tratt fruit, *Annona squamosa*, wax apple, *Chaenomeles speciosa* seeds, and *Momordica charantia*, significantly reduced the activities of α-glucosidase or α-amylase, which showed great potentials in slowing starch enzymatic digestion (19, 91–99). The structure-function of polysaccharides toward enzymes activity has been not revealed yet.

Accordingly, non-starchy polysaccharides lowered starch digestion rate and extent by four different ways: (i) interacted with starch granules and restricted starch disruption during food processing (77–80); (ii) increased systems viscosity and in turned restricted enzymes diffusion onto starch molecules (85, 86); (iii) formed matrix structure with starch and increased rigidity and ordered structures of starch-non-starchy polysaccharide complexes (82, 88, 89); (iv) interacted with α-glucosidase or α-amylase and reduced enzymes activities (91).

## Polyphenols slow starch digestion

**Figure 2:**
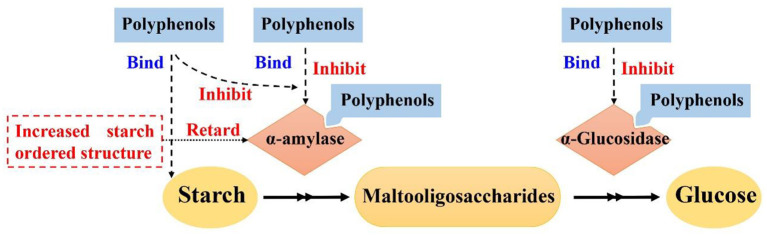
Mechanism scheme for effects of polyphenols on starch digestion. Figure was adapted from Sun and Miao (16).

**Figure 3:**
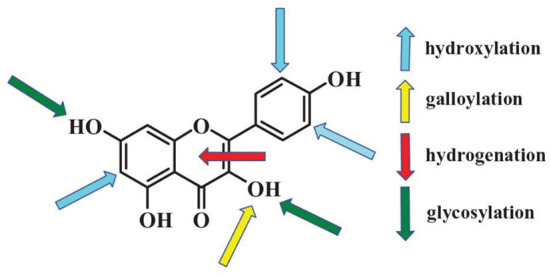
Flavonoids key sites that affecting activities of α-glucosidase. The *up arrows* and *down arrows* represent increasing and reducing the inhibition activity, respectively. Figure was collected from Xiao et al. (102).

Effects of polyphenols on starch digestion are schematically shown in Figure 2. α-amylase and α-glucosidase are two key enzymes for starch digestion. Accordingly, starch digestion could be significantly lowered through reducing activities of α-amylase and α-glucosidase. Tea polyphenols, flavonoids, phenolic acids, and tannins significantly reduced activities of α-amylase and α-glucosidase, which showed great potentials in mitigating starch digestion as summarized in previous reviews (16, 100–102). Polyphenols with different structures showed great differences in inhibition of activities of α-amylase and α-glucosidase. Effects of flavonoids structures on the inhibitory activity of α-glucosidase is schematically shown in Figure 3. The hydroxylation and galloylation of flavonoids improved the inhibitory activity, while the glycosylation of hyroxyl group and hydrogenation of the C2=C3 double bond on flavonoids, and the mono-glycosylation of chalcones reduced the inhibition (102). Cooperating polyphenols into starchy foods systems can remarkably slowed starch enzymatic digestion.

Polyphenols could interact with starch and form ordered starch structures, and in turn, lowering starch digestion as shown in Figure 2. It has been reported that polyphenols could interact with starch and promote the formation of starch ordered structures (103–110). Tea polyphenols, sorghum phenolic compounds, gallic acid could non-covalently interact with starch to form ordered structures for lowering starch digestion extent (103, 105, 106, 111–114). V-type crystals are the structures that are highly resistant to enzymatic digestion (8). Tannins, proanthocyanidins, and longan seed polyphenols interacted with starch and formed V-type crystals, which significantly lowered starch digestion rate and extent (105, 107–109, 111). Proanthocyanidins with a higher degree of polymerization had stronger abilities to interact with starch and form more V-type crystals (107, 114). Controlling the molecular weight of proanthocyanidins would be a promising pathway to control the formation of V-type crystals and thus starch enzymatic digestion (114). Hydrophilic polyphenols were hardly to form V-type with starch using conventional complexation method (e.g., high speed shearing) (115). However, high pressure homogenization could promote starch interaction with gallic acid and green tea polyphenols, thereby forming V-type crystals and short-range ordered structures for lowering starch enzymatic digestion (105, 112). In addition to V-type crystals, gallic acid might form “hamburger-like” structure of starch-polyphenol-starch complexes, which increased ordered structure of starch gel and lowered starch digestion extent (103).

## Other dietary compounds

In addition to the compounds discussed above, other dietary compounds such as NaCl and phytosterols also affected starch digestibility (70, 116, 117). NaCl would promote the formation of starch-lipid inclusion complexes, which would slow starch enzymatic digestion in a higher magnitude (70). Generally, retrograded starch showed a low digestibility due to its ordered structures (118). Co-crystallization with NaCl to produce single-helix amylopectin was regarded as a promising strategy to retard starch retrogradation (116), suggesting NaCl potentially increased digestibility of retrograded starch. Phytosterols, which showed robust abilities in lowering enzymes activities (117), indicating phytosterols could slow starch digestion significantly *via* inhibiting enzymes activities.

## Concluding remarks and future directions

This review summarized effects of dietary compounds including cell walls, proteins, lipids, non-starchy polysaccharides, and polyphenols on starch enzymatic digestion and their underlying mechanisms were discussed. Dietary compounds lowered starch digestion through three pathways: (i) retained starch ordered structures or formed ordered assemblies chaperoned with these dietary compounds; (ii) formed physical barriers and prevented enzymes from accessing/binding to starch; (iii) reduced enzymes activities. Cell walls, proteins, and non-starchy polysaccharides restricted starch disruption during hydrothermal treatment and the retained ordered structures limited enzymatic binding. In addition, they encapsulated starch granules and formed physical barriers for enzymes accessing. Proteins, non-starchy polysaccharides along with lipids and polyphenols interacted with starch and formed ordered assemblies. Non-starchy polysaccharides and polyphenols showed robust ability to reduce activities of α-amylase and α-glucosidase. Comparing with cell walls, protein, and non-starchy polysaccharides, lipids and polyphenols had stronger ability to slow starch digestion.

Food systems are relative complex with cell walls, proteins, lipids, non-starchy polysaccharides, polyphenols, vitamin, minerals, sugar, salts, etc. Dietary compounds might interact with each other and affect starch digestion in complicated pathways. How the complex dietary compounds affected starch digestion in real foods systems must be further investigated. Currently, effects of dietary compounds on starch digestion were interrogated *in vitro*. Dietary compounds would be digested and absorbed in the gastrointestinal tract and in turn affected starch digestibility. Starch *in vitro* digestibility may quite different to *in vivo* digestibility. Accordingly, *in vivo* glycemic response is the most important property of starchy foods. Further studies are needed to investigate the roles of dietary compounds on starch *in vivo* glycemic response. In addition, different groups of people such as children, athletes, middle- and old-aged human have different requirements for starch digestion rate and extent. Targeted structuring food structures and starch digestion behaviors *via* complexation with dietary compounds remains of interest.

## Author contributions

CC: conceptualization, literature analysis, reviewing, and editing. MS: reviewing and editing. YZ: supervision, writing, reviewing, and editing. BC: investigation, writing, and editing. YH: investigation and reviewing. MW: supervision, reviewing, and editing. All authors contributed to the article and approved the submitted version.

## Funding

The research was financially supported from project of the Scientific Research Innovation Program Xiyuanjiang River Scholarship of College of Life Sciences, Fujian Normal University (22FSSK004).

## Conflict of interest

The authors declare that the research was conducted in the absence of any commercial or financial relationships that could be construed as a potential conflict of interest.

## Publisher's note

All claims expressed in this article are solely those of the authors and do not necessarily represent those of their affiliated organizations, or those of the publisher, the editors and the reviewers. Any product that may be evaluated in this article, or claim that may be made by its manufacturer, is not guaranteed or endorsed by the publisher.
